# Physical therapy and anesthetic blockage for treating temporomandibular disorders: A clinical trial

**DOI:** 10.4317/medoral.17491

**Published:** 2012-12-10

**Authors:** Mirella M. Nascimento, Belmiro C. Vasconcelos, Gabriela G. Porto, Greiciane Ferdinanda, Cyntia M. Nogueira, Ronaldo C. Raimundo

**Affiliations:** 1 Postgraduate Student of the PhD program in Oral and Maxillofacial Surgery, University of Pernambuco (Recife-Brazil); 2 Senior Lecturer in Oral and Maxillofacial Surgery. Director of the Master and PhD programs in Oral and Maxillofacial Surgery, University of Pernambuco (Recife-Brazil); 3 Specialist in Oral Rehabilitation

## Abstract

Purpose: the aim of this study was to evaluate the use of physical therapy and anesthetic blockage of the auriculotemporal nerve as a treatment for temporomandibular joint disorders. Methods: the sample comprised of twenty patients with a diagnosis of disc displacement with/ without reduction and arthralgia according to the Research Diagnostic Criteria for Temporomandibular Disorders (RDC/TMD Axis I Group IIa, IIb and IIIa). Ten patients (group 1) underwent a cycle of eight anesthetic blockages of the auriculotemporal nerve with injections (1 per week) of 1 ml of bupivacaine 0.5% without vasoconstrictor for 8 weeks. The other 10 patients (group 2) received anesthetic blockage and physical therapy (massage and muscular stretching exercises). After the end of treatment all patients were evaluated at baseline, 1st week, 4th week and 2 months. The t-Student and F (ANOVA) tests were used for statistical analysis, with a significance rate of 5%. Results: there was a significant difference when both groups were compared according to VAS score (p=0.027). There was no significant difference for the other variables: MMO and jaw protrusion. Conclusion: the anesthetic blockage and physical therapy, when used together, are effective in the reduction of pain in patients with TMD.

** Key words:**Temporomandibular joint disorders, physical therapy, physiotherapy and nerve block, local anesthetic, bupivacaine.

## Introduction

Temporomandibular disorder (TMD) is a term used to describe a number of related disorders involving the temporomandibular joint (TMJ), masticator muscle, and occlusion (1). The condition affects approximately 10% of the population (2), although TMD is not considered a public health problem (3).This is a relatively common condition occurring at any age with a predilection for women at early adult ages (4), being 1.5-2 times more prevalent in women than in men and 80% of the patients treated for this disorder are women (5).

TMD is the most common cause of orofacial pain of non-dental patients (6). Its etiology is multi factorial and still poorly under-stood (1). A variety of possible etiological factors have been studied, such as occlusion (7), depression, stress and anxiety (8,9).

Treatment for TMDs has been discussed in the literature for at least two centuries but treatment options have been only established during the last two decades. Disagreement and controversy remain among those who are active in diagnosing and treating TMDs (10).

The therapeutic methods described in the literature for these disorders include: physical therapy, occlusal appliance, biofeedback, pharmacotherapy, transcutaneous electrical nerve stimulation, psychological therapy (cognitive behavioral therapy) and surgery for joint disorders. Among the methods mentioned above, the physical therapy has been effective for most patients with TMD, especially for painful joint and limitation of mandibular movements (10-14). Even though the anesthetic blockage of the auricular nerve has not been studied for treating TMD, there is some evidence of anesthetic blockage for reducing shoulder pain an orthopedic (15-17). So it is expected that the anesthetic blockage will diminish the pain leading to a better functional performance of the joint, which enables its nutrition, waste removal and lubrication helping the joint recovery (18).

Therefore the aim of this study was to use physical therapy and anesthetic blockage of the auriculotemporal nerve, specifically the anesthetic blockage for the first time in literature as an option for treating patients with temporomandibular joint disorders.

## Material and Methods

-Study design

A convenience sample was composed of twenty patients (28 TMJs). A randomized clinical blind study was carried out at the Center of Clinical Research in Oral-Maxillofacial Surgery at the School of Dentistry of Pernambuco – University of Pernambuco, Brazil from March to December 2008. All participants agreed to answer the questionnaire and signed the informed consent term. The study received the approval of the Ethics in Research Committee (Process n° CEP/UPE: 209/07 – Register number CAAE: 0117.0.097.000-07).

The patients were diagnosed with temporomandibular joint disorders using the Research Diagnostic Criteria for Temporomandibular Disorders (RDC/TMD). Before being treated, one examiner (GGP) performed the screening, history-taking and clinical examination and evaluated the patients after treatment. Another examiner was an orofacial pain specialist (CMN) calibrated according to the RDC ⁄TMD selected the patients. A third (MMN) and fourth (GF) examiners applied anesthetic blockage of the auriculotemporal nerve and physical therapy. Both examiners were blinded to group assignment.

Patients were selected according the following to inclusion criterias: both sex above 18 years of age, patients with disc displacement and arthralgia (group II and IIIa - RDC / TMD) and with scores from 3 to 9 of Visual Analog Scale for pain assessment. Exclusion criteria were previous treatment with pharmacotherapy, previous use of occlusal appliances, symptoms related to disease in other parts of the stomatognathic system (e.g. toothache, neuralgia), pain due to systemic disease (e.g. rheumatoid arthritis), fibromyalgia and history of psychiatric disorders.

The patients were randomized in two groups for treatment. Ten patients (group 1 – positive control group) were treated with a series of eight anesthetic blockages of the auriculotemporal nerve with injections (1 per week) of 1 ml of bupivacaine 0.5% without vasoconstrictor for 8 weeks. The other 10 patients (group 2 – experimental group) received the anesthetic blockage and physical therapy (massage and muscular stretching exercises). Interincisal distance at maximal mouth opening and jaw protusion was recorded and the patient was asked to quantify the pain using a visual analog scale (VAS). All assessments were repeated by the same physician at baseline (preoperative), 1st week, 4th week and 2 months after the last injection. The Kolmogorov-Smirnov test was used to determine the data was normally distributed. The t-Student test and the F test (ANOVA) were used for statistical analysis with 5% of significance rate.

-Injection Technique

The condyle was palpated with the tip of the index finger to reach the point of introduction of anesthesia, while the patients were instructed to open and close the mouth. Then the patients were asked to open the mouth as wide as possible to find the condylar neck, approximately 1-1.5 cm below the tragus (19,20). The needle is passed through the skin about 4 mm in front of the tragal cartilage and directed inward and forward until the condyle is felt by the need tip that passed behind the neck of the condyle for a further penetration of 1 cm, where 1 ml of solution is deposited in the region of the auriculotemporal nerve. The anesthetic was deposited at this point after negative aspiration (20).

-Physical Therapy Technique

The physical therapy was performed by a physical therapist after the anesthetic blockage in group 2. The techniques comprised in mobilizing the joint, passive traction and translation movement, these movements were carried out in every all direction (left, right and anterior). This mobilizing procedure was accompanied by massage exercise of the jaw elevator muscles (temporal and masseter) (21). All patients were treated for 30 min once a week by the same physical therapist (GF). After the first 3 appointments, the patients were thought to do all the exercises at home, 3-4 series of 2 minutes each in front of a mirror.

After 8 weeks, the physical therapy and the anesthetic blockage were suspended and the patient were told not do the exercises at home. After 2 months the patients were revaluated.

## Results

All patients included in this study were followed for a period of 2 months. Few complications were observed due to injection, such as temporary anesthesia of the facial nerve, hematoma at the injection site and positive aspiration ( Table 1). Detailed data about patient demographics, clinical presentations and outcomes are presented in  Table 2.

**Table 1:**
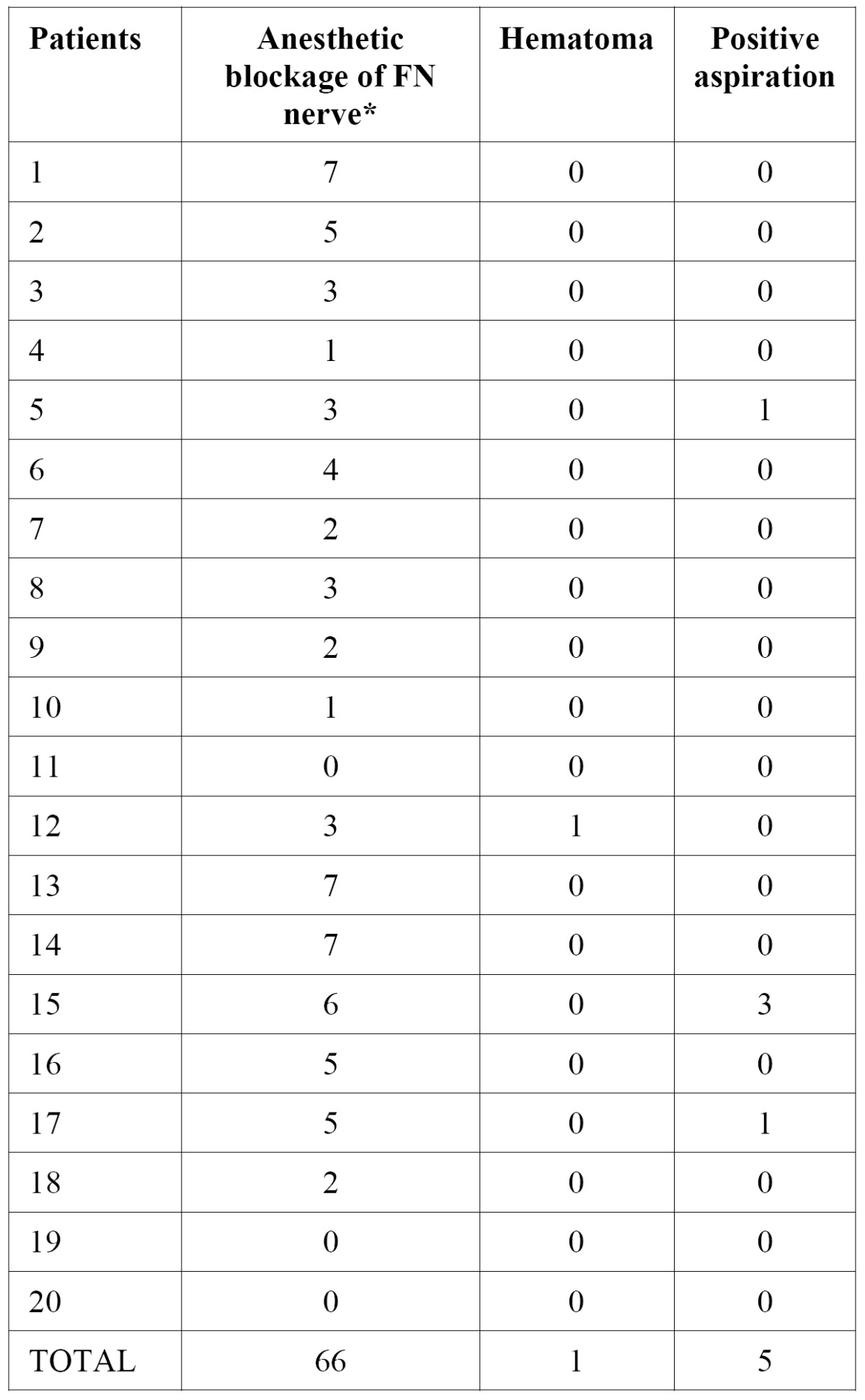
Distribution of subjects according to the anesthetic blockage of the AT nerve, hematoma and positive aspiration.

**Table 2:**
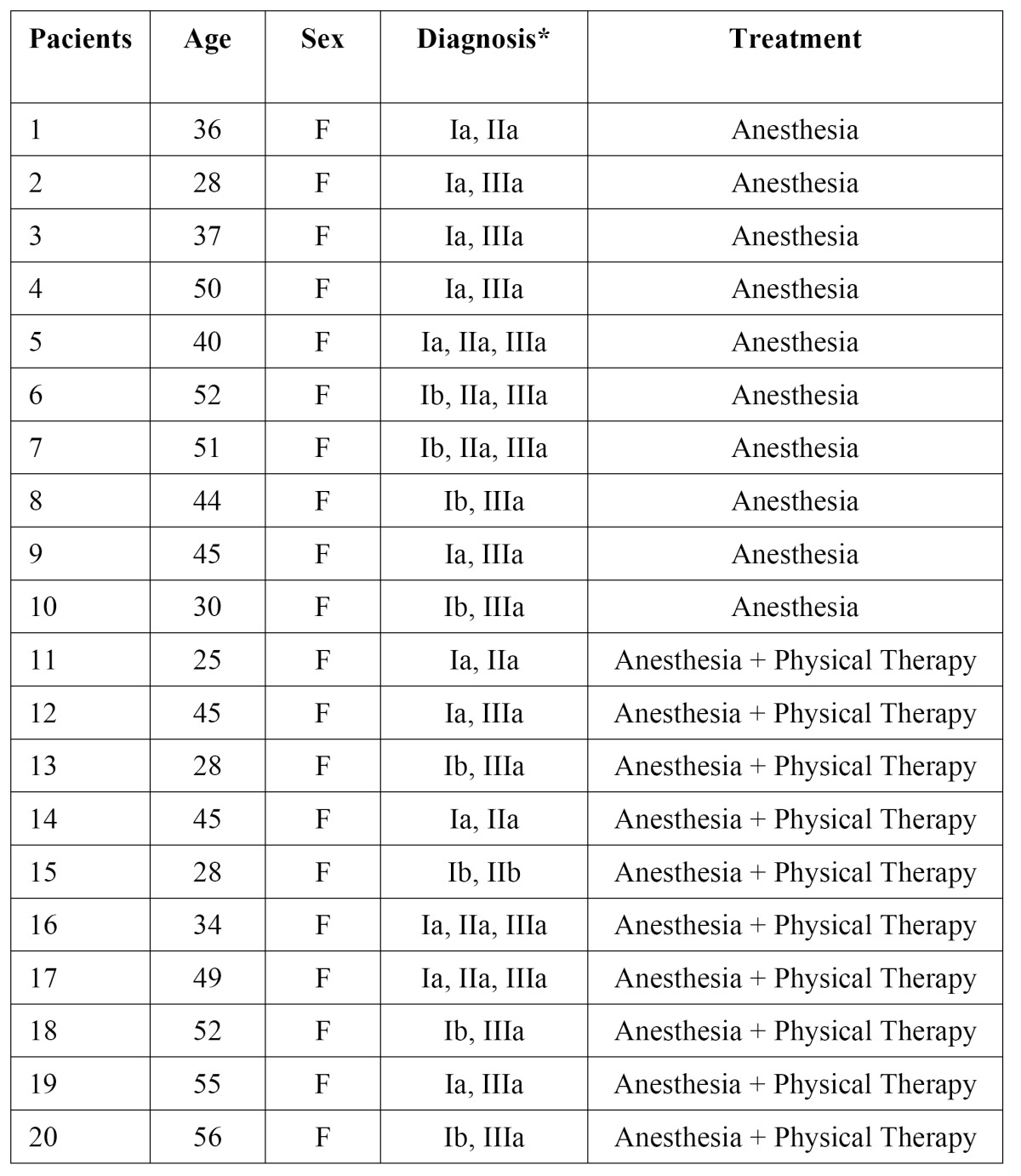
Demographic data of the patients.

The mean and standard deviation values of the VAS scores, maximum mouth opening and jaw protusion measures according to the times of evaluation (baseline, 1st and 4th week and 2 months) for both groups are shown in  Table 3.

**Table 3:**
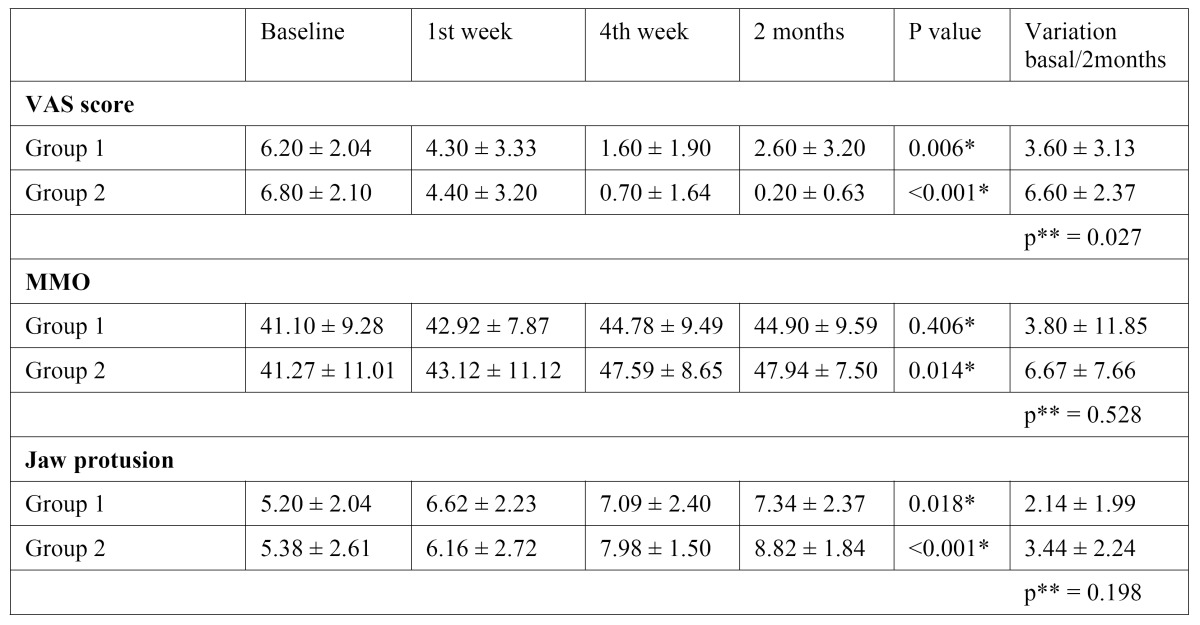
Mean and standard deviation values of the VAS scores, MMO and jaw protusion measures according to the times of evaluation.

## Discussion

Based on orthopedic techniques for the treatment of adhesive capsulitis using blockage of the supra-scapular nerve associated with physiotherapy, this paper hypothesized that the use of anesthetic blockage of the auriculotemporal nerve and physical therapy could be applied as a treatment for temporomandibular joint disorders (15-17,22), since a painless joint may lead to a reestablishment of its function, which enables its nutrition, waste removal and lubrication (18). No study has been published using this study model in the TMJ until the present date.

In this study, it was observed that the sample consisted of female patients, but not on purpose. Epidemiological studies make clear that female patients of reproductive age are most affected with TMD and seek more treatment than the males (23-25). This observation is confirmed in this trial. With respect to age, the sample consisted of patients aged 25 to 56 years, average 41.5 ± 10.1 years. Unlike the epidemiological study of Manfredini et al. (26) that observed that patients with a mean age were lower 38.8 ± 15.7 years (aged 18 to 82 years).

The manual therapy is described in the literature for the treatment of patients with joint disorders (13). In this work, the physical therapy consisted of massage with stretching exercises active/passive and resistance to masticatory muscle. Participants were instructed to perform stretching exercises at home. During the study there was no difficulty in adhering the patient to the exercises at home. After 8 weeks treatment was stopped, including the home exercises. However, during the two months of follow-up it was not possible to prevent the patient to perform the exercises at home in are of pain.

A total of 28 joints were treated during eight weeks, with 224 anesthetic nerve blockages of the auriculotemporal nerve. Several studies reported findings of the anesthetic blockage of this nerve to perform artrocenthesis and arthrography, but none has studied the anesthetic blockage as an option for treating TMD´s, which makes only possible the discussion about its complications. One of these studies was made by Dolon et al. (20) who made 60 anesthetics blockages in 50 patients to perform arthrography and artrocentese. Out of these, 20% of patients (10/50) reported discomfort at the beginning or at end of the procedure and 8% (4/50) reported pain during the procedure. The same authors stated that the nerve blockage of the auriculotemporal nerve was unsuccessful when the blockage was performed above the place of the described technique. In this study, none patients in group 2 reported discomfort during the physical therapy period.

As complications of the procedure in this study, 29.4% (66/224) of the patients had temporary facial nerve paralysis, 0.44% (1/224) hematoma and 2.23% (5/224) positive aspirations. Although studies of Dolon et al. (20) showed complications such as: 2% (1/50) subcutaneous hematoma, 14% (7/50) of zigomaticotemporal paralysis and 24% (12/50) of the orbicular muscle paralysis and no positive aspiration. The high rate of paralysis of the facial nerve can be justified by the close contact of nerve facial with the auriculotemporal nerve observed in others studies (27,28).

In group1 (anesthetic blockages of the auriculotemporal nerve), there was significant decrease in average pain levels, at two months of follow up these patients suffered less pain in the joint. In group 2 (anesthetic blockage and physical therapy), it was different because the average pain levels decreased in during the clinical trial. The results of the treatment with anesthetic blockage and physical therapy tend to support similar results in the literature, specifically a study using bupivacaine blockage of the suprascapular nerve and motion exercises of shoulder (15). The other studies that used only physical therapy presented decrease of pain after treatment of temporomandibular joint disease (12,13).

All patients of both groups also demonstrated an improvement in their maximum mouth opening and jaw protusion. The improvement of mandibular function is expected because the treatment leads to a better lubrication of the joint. NITZAN

Meanwhile, another study of Nicolakis et al. (13) did not achieve success with the exercises therapy applied in 20 patients, until the end of the study seven of them had returned with maximum mouth opening worsening. However it is important to mention that the patients used in this study were diagnosed with disc displacement without reduction. Unlike the study of Nicolakis et al. (13), in this paper the patient who had disc displacement without reduction (n=1, group 2) did not have a maximum mouth opening decrease, instead there was an increase from 22 to 42 mm. In this case, it is believed that the treatment with anesthetic blockage and physical therapy did not return the disc to its proper place, but there has been an adaptation of the joint structures.

## Conclusion

The results of this study support that anesthetic blockage of the auriculotemporal nerve and physical therapy are effective in the reduction of the patients’ pain with temporomandibular joint disorders. The authors suggest that the anesthetic blockage of the auriculotemporal nerve is a technique that may be used as a tool for the diagnosis and treatment of acute pain of the joint. In addition is a noninvasive, low-cost treatment and has a low rate of complications.
